# Lhermitte-Duclos Disease: A Case Series

**DOI:** 10.7759/cureus.44326

**Published:** 2023-08-29

**Authors:** Gonzalo Monjarás-Romo, Lilian Zavala-Romero, Maria Fernanda Tejada-Pineda, Juan Marcos Meraz-Soto, Daniel Ballesteros-Herrera, Jesús Cienfuegos-Meza, Roberto J Alcazar-Felix, Sergio Moreno-Jiménez

**Affiliations:** 1 Radiosurgery Department, National Institute of Neurology and Neurosurgery “Manuel Velasco Suárez”, Mexico, MEX; 2 Pathology Department, National Institute of Neurology and Neurosurgery “Manuel Velasco Suárez”, Mexico, MEX; 3 Neurosurgery-Radiosurgery Department, American British Cowdray Medical Center, Mexico, MEX

**Keywords:** neurosurgery, cowden syndrome, posterior fossa, dysplastic cerebellar gangliocytoma, lhermitte-duclos disease

## Abstract

Lhermitte-Duclos disease (LDD), or dysplastic cerebellar gangliocytoma, is a rare benign tumor characterized by unilateral hemispheric cerebellar expansion. It is linked to mutations in the phosphatase and tensin homolog (PTEN) gene, which inhibit the phosphatidylinositol-3'-kinase pathway, leading to increased cell division and defective neuronal migration. This study aims to compare the clinical, radiological, histopathological, surgical resolution, and follow-up characteristics of reported cases of this rare condition. An in-depth search of LDD patients' clinical records at our institute between 2003 and 2023 was conducted, in addition to a systematic literature review on PubMed. Three patients with a diagnosis of LDD were found. Cerebellar abnormalities, varying headaches, and visual impairment were all present clinically. On T2 in the posterior fossa, all three MRI scans displayed the typical hyperintense parallel streak appearance. The histopathological report showed that large ganglion cells had replaced the granular layer, Purkinje cells had degenerated, the molecular layer had become hyper-myelinated, and synaptophysin and chromogranin were positive. Partial tumor resection and avoiding intracranial hypertension were the main goals of treatment. Genetic follow-up was conducted for all three patients. Neurosurgeons must be aware of LDD to provide close genetic monitoring despite the benign nature of the tumor because of its link to Cowden syndrome and elevated risk of cancer in other organs.

## Introduction

Lhermitte-Duclos disease (LDD), or dysplastic cerebellar gangliocytoma, is a rare benign tumor characterized by unilateral hemispheric expansion of the cerebellum [1]. This tumor is characterized by the abnormal growth of ganglion cells, which generally help regulate the activities of the cerebellum. In most cases, it is associated with mutations in the tumor suppressor gene PTEN, which inhibits the phosphatidylinositol-3'-kinase (PI3K) pathway and ultimately leads to increased cell division and defective neuronal migration [1,2].

Symptoms of the disease can vary widely but may include headaches, difficulty with balance and coordination, and problems with vision or hearing [1]. Diagnosis may be made through various tests, including imaging studies such as magnetic resonance imaging (MRI) and computed tomography (CT) [3]. Treatment options for LDD depend on the size and location of the tumor and the severity of symptoms. Surgery may be recommended to remove the tumor and relieve pressure on surrounding structures [1,4].

In this article, we present three cases retrieved from our institution's database dating from 2003 to 2023.

## Case presentation

Case 1

**Figure 1:**
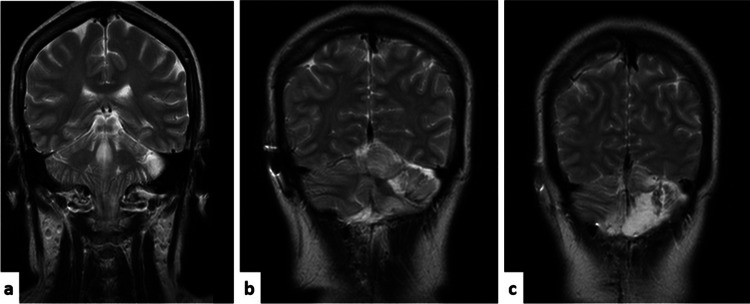
Coronal MRI with a cerebellar left hemispheric lesion hyper-intense in T2 signal, with thinning of white matter (hyperintense) and thickening of gray matter (isointense) with a striped appearance. This is an original figure.

In 2012, a 44-year-old female, with a family history of unspecified cerebral tumors, was brought to the outpatient clinic due to cephalea and right-sided gait lateropulsion, which she had experienced for the last seven years. A CT scan revealed hydrocephalus. Thus, a ventriculoperitoneal shunt was placed. In September 2013, she started experiencing cephalea and dizziness. MRI displayed a left cerebellar hemispheric lesion with a “tiger stripe” appearance (Figure 1).

**Figure 2:**
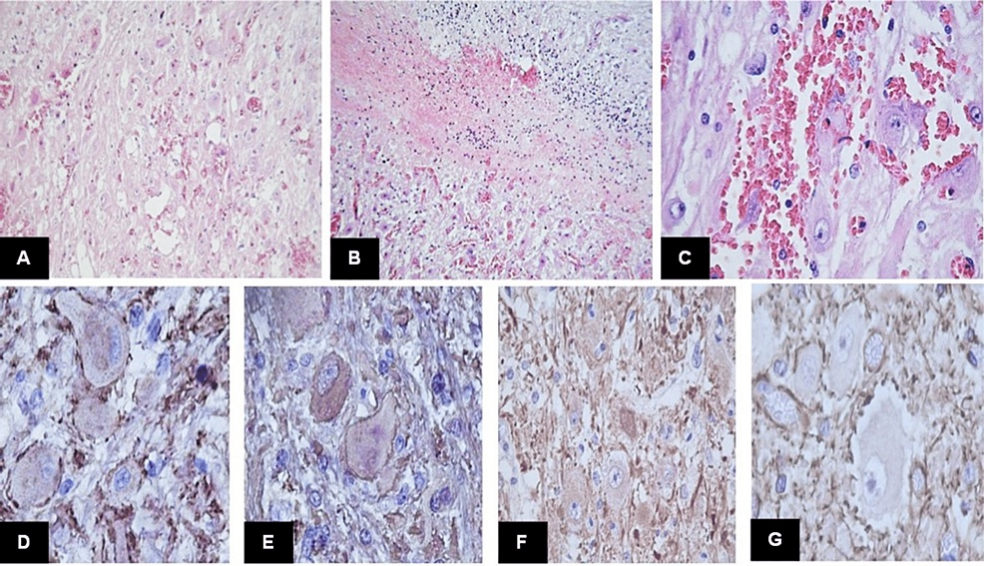
Histological features of Case 1. A) Large dysplastic ganglion cells with rich eosinophilic cytoplasm. B) Dysplastic ganglion cells replace and infiltrate the cerebellum cortex. C) Ganglion cells showed fine chromatin and prominent nucleolus with hemorrhage. D) Neuron-specific enolase was positive in ganglion cells (NSE; 40x). E) Dysplastic ganglion cells also showed immunoreactivity for synaptophysin (40x). F) Neurofilaments were in normal configuration inside ganglion cells (NF, 40x). G) GFAP showed reactivity in astrocytes surrounding ganglion cells (40x). This is an original figure.

She was scheduled for tumor resection with a histologic diagnosis of astrocytoma. Then, she was referred to our hospital for adjuvant management and follow-up. The physical examination was normal. An additional review of histologic findings was performed, and dysplastic cerebellar gangliocytoma was diagnosed (Figure 2).

Case 2

**Figure 3:**
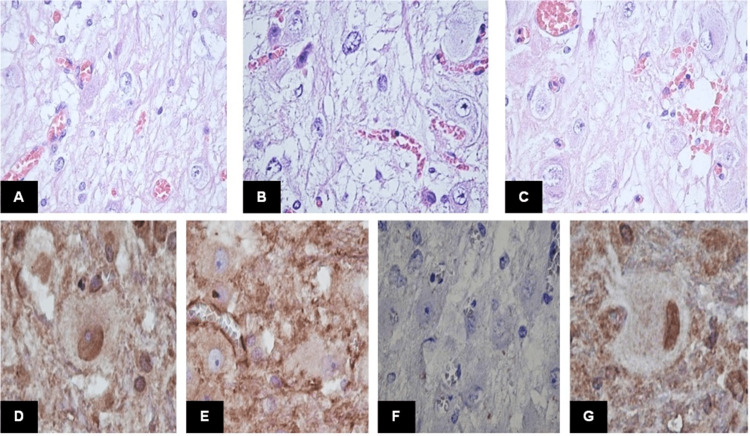
Histological features of Case 2. A) This case showed smaller dysplastic ganglion cells (H&E; 40x). B) Stromal changes with vacuolization were predominant (H&E; 40x). C) Some ganglion cells showed eosinophilic glassy cytoplasm (H&E; 40x). D)AKT cytoplasmic expression was found in ganglion cells (40x). E) AKT1 was found in the ganglion cell membrane, and the nucleus was negative (40x). F) PTEN immunoexpression was negative (40x). G) Phosphorylated PTEN was found positive in ganglion cell nuclei suggesting PTEN overexpression (40x). This is an original figure.

A 36-year-old female came to the outpatient clinic after 11 years of experiencing biparietal oppressive cephalea that worsened with Valsalva and hypoesthesia of the right side of her face. She had a family history of hamartomatous gastric tumor and a personal history of multinodular goiter, which was monitored by a neuroendocrinologist. Physical examination found bilateral papilledema, left dysmetria, and dysdiadochokinesia. A non-contrast CT showed acute hydrocephalus and a left cerebellar lesion. A ventriculoperitoneal shunt was placed. MRI confirmed a left cerebellar lesion with a “tiger stripe” appearance that compressed the brain stem. Tumor resection was scheduled, and a subtotal resection was achieved. Intraoperative findings described an infiltrating lesion with no clear edges that distorted cerebellar tissue. She was discharged five days after the resection with a good functional status. Histopathology reported a dysplastic cerebellar gangliocytoma (Figure 3).

She experienced a recurrence five years later that required valvular replacement and new resection. Follow-up revealed a left cerebellar syndrome and ataxia.

Case 3

**Figure 4:**
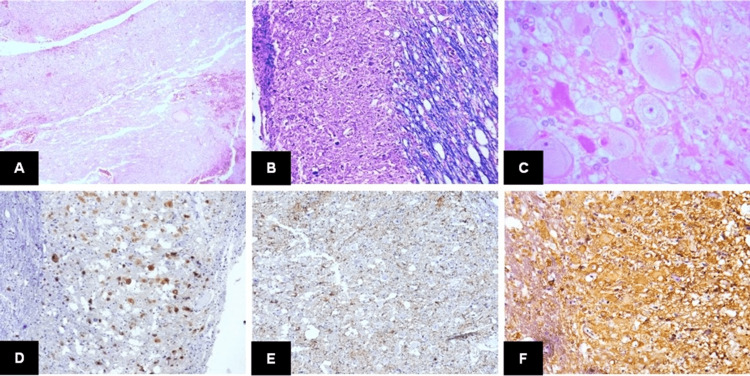
Histological features of Case 3. A) Cerebellar cortex replaced by tumor (H&E; 4x). B) Dysplastic ganglion cells infiltrating the molecular layer; note the abnormal myelination of white matter (Kluver-Barrera stain; 10x). C) Dysplastic ganglion cells and vacuolated stroma (H&E; 40x). D) Ganglion cells were reactive to NeuN (NeuN; 10x). E) Reactive astrocytes surrounding ganglion cells (GFAP; 10x). F) Ganglion cells were reactive to neuron-specific enolase (20x). This is an original figure.

A 53-year-old man, diagnosed with hypertension four years ago, was treated with losartan and hydrochlorothiazide. Without a previous history of brain disease presented himself to the ophthalmologist with nystagmus. He was treated with timolol, but symptoms worsened, adding ataxia, vertigo, and occipital cephalea rated 8/10 on the visual analog scale (VAS), so he arrived again with an ophthalmologist who referred to a neurologist, who indicated image studies. Thus, he was referred to our institution. An MRI was performed at his arrival, and obstructive hydrocephalus and a right cerebellar lesion were found. Thus, the patient was referred to the emergency department. His physical examination showed nystagmus, dysmetria, dysdiadochokinesia, and ataxia. Frontal cephalea worsened with the Valsalva maneuver, presenting occipital cephalea. Strength was conserved in all extremities, and muscle stretch reflex was +++/+++++ in every extremity. The patient was admitted to the hospital with right cerebellar syndrome and hydrocephalus diagnosis. Two days later, he underwent surgery to achieve partial resection; cortectomy was performed in the right cerebellar hemisphere, and going deeper with Mallis and Surgicel, the lesion was partially removed. The patient was discharged three days after for home care. Histopathological examination confirmed a dysplastic gangliocytoma of the cerebellum (Figure 4).

The patient experienced persistence of the symptoms after four weeks, so he underwent a reintervention without any complications during the postoperative phase and was discharged after three days. The lesions were analyzed by neuropathologists at the institution, and the genetic analysis confirmed a positive result for LDD.

## Discussion

LDD is a rare condition, with approximately 230 cases reported in the literature, typically affecting patients aged between 30 and 50. There is no sex preference. LDD is a rare brain tumor in the posterior fossa, described as a hamartomatous lesion due to abnormal development and unilateral hemispheric expansion of the cerebellum.

This disease has been described in different terms throughout history, including gangliocytoma, ganglioneuroma, Purkinjeoma, granule-molecular hypertrophy of the cerebellum, and granule-cell hypertrophy.

The nature of LDD, pathogenesis, and the exact genetic basis are unknown. However, a germline mutation of phosphatase and tensin homolog on chromosome 10q23 is considered widely as the underlying defect [2]. This aspect is related to PTEN hamartoma tumor syndrome (PHTS), a spectrum of disorders caused by mutations in the phosphatase and tensin homolog (PTEN) gene located on this chromosome. Loss of PTEN function leads to unimpeded phosphatidylinositol-3'-kinase (PI3K) activity and PI3K-driven cell division [5]. Individuals with PHTS develop benign hamartomas in various locations and have an increased risk of developing malignant diseases. It is widely accepted that two disorders that compose this spectrum, Cowden syndrome and Bannayan-Riley-Ruvalcaba syndrome, are allelic conditions [6,7]. Several associated conditions have been described, including Cowden disease, Lhermitte-Duclos-Cowden syndrome or COLD syndrome, disorders of cortical formation with megalocephaly, gray matter heterotopia, polymicrogyria, polydactyly, polydactyly hydromyelia macroglossia, localized gigantism, and leontiasis osseous [8].

Although many cases of LDD have not been studied for genetic alterations, some series describe patients with this disease who previously had breast cancer or a history of multiple neoplasms, including colon carcinoma and polyps [9]. In our series, these associations were also present. Case 1 referred to a family history of brain tumors, and Case 2 referred to hamartomatous gastric tumors with familial evidence.

The classical description of this disease in MRI is a non-enhancing mass in the posterior fossa that appears hypointense on T1 and hyperintense on T2-weighted images. It appears with linear parallel striae, giving the cerebellum a “tiger stripe” appearance. Some authors have proposed that the regions of T1 and T2 prolongation correspond to the central portion of the folia; their changes in signal intensity may be due to demyelinated central white matter. On the other hand, isointense areas represent the outer segment of the molecular layer [9]. Klisch et al. described restricted diffusion, high vascularity, and preserved blood-brain barrier permeability on PWI as usual findings [3]. Restricted diffusion can be explained by increased cell density. Regarding functional imaging evaluation, previous studies used nuclear energy, xenon-CT, and PWI, which have shown an elevated metabolic rate characteristic of tumors with growth potential [3,10]. However, MR spectroscopy usually reports normal Cho/Cr (choline/creatine) ratios, demonstrating the absence of significant membrane turnover, unlike what is commonly seen in aggressive proliferative neoplastic processes.

This particular appearance at MRI may be sufficiently unusual to suggest the diagnosis of LDD preoperatively, and it is also useful to determine the margins in surgical planning. A differential diagnosis of LDD is cerebellar infarct because it is visualized as a non-enhancing lesion with mass effect. However, an acute clinical presentation and changes in appearance on subsequent MR images obtained over days or weeks make confusion with LDD unlikely [9].

Complete tumor removal is not usually performed due to the technical difficulty associated with the tumor’s location. Additionally, the pathogenesis of these tumors results from hypertrophy of the cerebellum's granular layer. Therefore, complete removal would also entail the removal of the cerebellum itself. The dysplastic lesion grows very slowly; initial treatment usually involves resolving secondary hydrocephalus. Subsequently, surgical resection is the curative option, with some reported cases of recurrence [6].

Macroscopically, DGC shows thickened folia with surface pallor or yellow discoloration. In some cases, white matter cavitation can be found. The histological hallmark of DGC is an enlargement of cerebellar cortical thickness by large dysplastic ganglion cells [11]. These ganglion cells variably replace granular cells and infiltrate the molecular layer, constituting a heterogeneous neuronal population. There are large, polygonal neurons with prominent nucleoli and abundant mitochondria. They exhibit scarce Nissl substance, and cytoplasmic processes are filled with intermediate filaments and microtubules. However, the predominant ganglion cell type is medium to large-sized with hyperchromatic nuclei, fewer mitochondria, and more free ribosomes [12]. These cells have multipolar and tangled processes and can be found in the subpial space of the molecular layer, suggesting disrupted neuronal migration during cerebellar development. Another consistent histological finding is the abnormal myelination of the molecular layer, where fibers run parallel in the deeper portion and perpendicular to the pial surface. The cerebellar white matter often shows vacuolation. Additional findings include microcalcification and dense capillary networks. The dysplastic ganglion cells are positive for synaptophysin and NeuN, exhibiting a neurofilament expression pattern similar to granular cells. However, a subset of cells shares the immunohistochemical features of Purkinje cells [12]. In this series, all cases showed variable substitution of the cerebellar cortex by dysplastic ganglion cells, with prominent myelinated fibers in the subpial space and vacuolization of the white matter. Only one case showed calcifications.

## Conclusions

Recognizing LDD is of paramount importance, despite its infrequent occurrence. Although this condition may be rare, understanding its characteristics and presentation is crucial for early detection and appropriate management. Although noncancerous, LDD is associated with Cowden syndrome, a genetic disorder characterized by multiple noncancerous growths and an elevated risk of certain cancers in other organs. Due to this association, regular monitoring and genetic counseling are necessary to identify at-risk individuals and implement appropriate screening measures. The primary treatment objective for LDD is the surgical removal of the tumor and alleviation of intracranial hypertension. By recognizing the unique features of LDD and its potential implications, healthcare professionals can ensure early diagnosis, prompt intervention, and comprehensive care for individuals affected by this rare condition.
